# Assessment of Pharmacotherapy Modifications During the Treatment of Episodes of Acutely Decompensated Heart Failure: The HEROES Study

**DOI:** 10.3390/jcm14227980

**Published:** 2025-11-11

**Authors:** Agata Galas, Robert Morawiec, Agnieszka Kapłon Cieślicka, Katarzyna Byczkowska, Witold Furmanek, Adrian Stefański, Beata Wożakowska-Kapłon, Dominika Klimczak-Tomaniak, Piotr Hamala, Anna Furman-Niedziejko, Jarosław Drożdż, Paweł Krzesiński

**Affiliations:** 1 Department of Cardiology and Internal Diseases, Military Institute of Medicine—National Research Institute, 04-141 Warsaw, Poland; pkrzesinski@wim.mil.pl; 2 2nd Department of Cardiology, Medical University of Lodz, 92-213 Lodz, Poland; 3 1st Cardiology Department, Medical University of Warsaw, 00-575 Warsaw, Poland; 4 Heart Failure and Transplantology Department, National Institute of Cardiology—National Research Institute, 04-628 Warsaw, Poland; kbyczkowska@ikard.pl; 5 Institute of Heart Diseases, Wroclaw Medical University, 50-368 Wroclaw, Poland; 6 Department of Hypertension and Diabetology, Faculty of Medicine, Medical University of Gdansk, 80-210 Gdansk, Poland; adrian.stefanski@gumed.edu.pl; 7 1st Clinic of Cardiology and Electrotherapy, Swietokrzyskie Cardiology Center, Jan Kochanowski University of Kielce, 25-369 Kielce, Poland; bw.kaplon@poczta.onet.pl; 8 Department of Cardiology, Hypertension and Internal Medicine, Medical University of Warsaw, 00-575 Warsaw, Poland; 9 1st Department of Cardiology, Medical University of Lodz, 92-213 Lodz, Poland; 10 Department of Coronary Disease and Heart Failure, Saint John Paul II Hospital, 31-202 Kraków, Poland; 11 Department of Emergency Medicine, Faculty of Health Sciences, Jagiellonian University Medical College, 31-008 Krakow, Poland

**Keywords:** heart failure, acute decompensated heart failure, hypervolemia, guideline-directed medical therapy, real-world observational study

## Abstract

**Background/Objectives:** Urgent hospitalization due to acutely decompensated heart failure (ADHF) is an unfavorable event in the trajectory of this disease. Patient condition during decompensation frequently limits opportunities to implement and optimize guideline-directed medical therapy (GDMT). To define the tasks of post-hospital care, it is essential to gain knowledge regarding the extent of GDMT implementation on the day of discharge after ADHF episodes. The purpose of this analisis was to evaluate GDMT changes during hospitalization due to ADHF, with a particular emphasis on patients with reduced ejection fraction. **Methods:** The analysis was conducted in a group of 262 patients hospitalized due to ADHF and with known left ventricular ejection fraction (LVEF). The HEROES study was a prospective, multi-center, observational study. **Results:** The mean age in the study group (196 men and 66 women) was 67.6 ± 14.6 years, with a mean LVEF of 33.9 ± 14.8%. Six patients died during hospitalization. In the analysis for the whole group (regardless of ejection fraction [EF]), ARNI (angiotensin receptor-neprilysin inhibitor)/ACEI (angiotensin-converting enzyme inhibitor)/ARB (angiotensin receptor blocker) use increased from 63.3% of the subjects at admission to 81.3% at discharge, beta-blocker use increased from 70.6% to 92.6%, MRA (mineralocorticoid receptor antagonist) use increased from 43.1% to 75.8%, and SGLT2i (sodium-glucose co-transporter 2 inhibitor) use increased from 30.1% to 75.0%. ARNI/ACEI/ARB therapy was optimized in 48.4% of the subjects, with optimization rates of 37.9%, 40.2%, and 44.1% for beta-blockers, MRAs, and SGLT2is, respectively. However, only 38 (22.0%) patients reached the level of treatment corresponding to “SGLT2i and ARNI/ACEI/ARB and betablocker and MRA in doses ≥ 50%”. **Conclusions:** In patients hospitalized due to ADHF in the HEROES study, the use of GDMT at discharge was significantly higher than at admission. In patients with reduced ejection fraction, GDMTs from all drug classes were prescribed to over 80% of patients. However, an insufficient number of patients attained high doses of GDMT, which emphasizes the need for effective dose up-titration in outpatient settings.

## 1. Introduction

Acute decompensated heart failure (ADHF) is a clinical syndrome of worsening of previously diagnosed heart failure (HF) or de novo heart failure (dnHF) [1,2] and is the main cause of HF hospitalizations. In most situations, HF deterioration is associated with hypervolemia [1,3]. Therefore, the primary action is diuretic treatment, whereas drugs that improve the prognosis are usually optimized with a delay due to several clinical limitations [1,2,4,5]. ADHF hospitalization signals disease progression and necessitates intensified guideline-directed medical therapy (GDMT). The STRONG HF study recommends an intensive treatment strategy of rapid up-titration of GDMT, as it improves quality of life and reduces the 180-day all-cause mortality rate and HF readmission rate when compared with usual care [5]. This method of GDMT up-titration is more difficult to implement in ADHF patients than in chronic HF (CHF) patients due to hypotension caused by excessive decongestion or transient deterioration of renal function and hyperkaliemia [6,7,8,9]. Urgent HF hospitalization is only the first step toward the implementation or up-titration of GDMT, and further outpatient care is of utmost importance [6,7,8,9,10,11]. Available data indicate that patients with CHF are often considered clinically stable, which contributes to a perceived lack of necessity for optimizing GDMT [12]. However, there is a scarcity of data regarding the optimization of GDMT in patients with ADHF. In the Polish clinical setting, analysis of the HEROES [HEart failuRe ObsErvational Study] registry demonstrates that while a substantial proportion of patients admitted for planned hospitalization undergo treatment optimization aimed at improving prognosis, the dosages of administered medications frequently remain significantly below recommended levels—only 22% of patients receive all GDMT in ≥50% of target doses [13].

To define the role of early post-hospital care in the Polish healthcare system, it is essential to present data regarding the extent of GDMT implementation on the day of discharge after an ADHF episode. In this context, we aimed to evaluate GDMT changes during hospitalization due to ADHF in participants in the HEROES study, with particular emphasis on patients with reduced left ventricular ejection fraction (LVEF).

## 2. Materials and Methods

**Figure 1:**
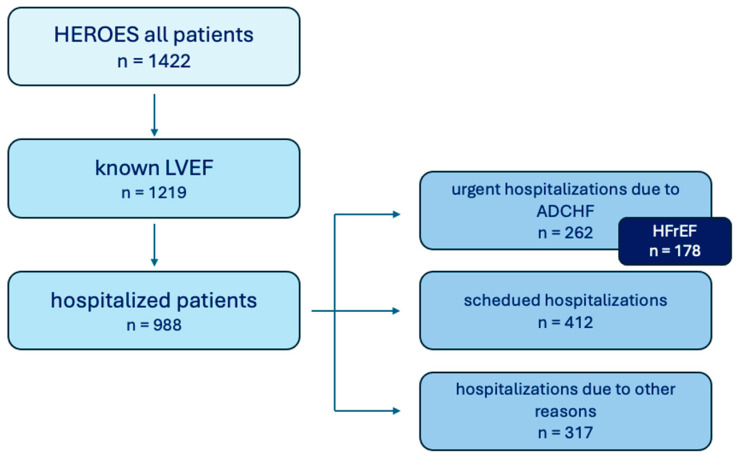
Population of HEROES Study.

The HEROES registry was a prospective, multicenter, observational study endorsed by the Polish Cardiac Society, which enrolled HF patients (with both normal and reduced LVEF) in hospital and ambulatory settings and was conducted by 41 Polish clinical centers [14]. There were no specific exclusion criteria, with the exception of the patient’s unwillingness to participate. The consent form for participation was distributed to all participants and signed. The study described the clinical status of HF patients, including tests performed, treatments given, and the quality of outpatient or hospital care provided in a representative national sample. The analysis was conducted in a group of 262 patients hospitalized due to ADHF and with a known LVEF who were selected from the 1422 participants of the HEROES study (Figure 1). Study protocol was approved by bioethical committee in Medical University of Lodz (decision number: RNN/316/20/KE from 20 December 2020 with further changes on resolution number KE/762/23 from 12 September 2023). The registry was funded by Polish Cardiac Society contract No. CRU 0120-KCKB-2023). The data were obtained from Polish clinical centers between April 2022 and March 2024.

The collected data included demographics, anamnesis with special attention to HF history, HF hospitalizations and etiology, comorbidities, and medications at admission and discharge. Moreover, the results of some diagnostics were noted, but only for those that were necessary during hospitalization for the physician’s evaluation. This may have included laboratory tests (blood count, liver function markers, serum creatine, serum sodium and potassium, and estimated glomerular filtration rate [eGFR]), transthoracic echocardiography with the assessment of LVEF, and a resting 12-lead electrocardiogram. The study design and methodology were previously presented in detail [14]. Artificial intelligence was not used in preparation of this manuscript.

In this analysis, we assessed the frequency of GDMT implementation, with a particular emphasis on the inclusion and dose increases of drugs that improve the prognosis of HFrEF patients. The criteria used to evaluate therapy optimization consisted of dose escalation, initiation of angiotensin-converting enzyme inhibitors (ACE-I), angiotensin receptor-neprilysin inhibitors (ARNI), beta-blockers, mineralocorticoid receptor antagonists (MRA), and sodium glucose cotransporter-2 inhibitors (SGLT2i), as well as switching from ACE-I or ARB to ARNI, or from ARB to ACE-I.

### Statistical Analysis

To describe the quantitative variables, we used the mean and standard deviation (SD) for normal distributions and the median (Me) and interquartile range (Q1–Q3) for non-normal distributions. The normality of the variables was verified using the Shapiro–Wilk test. For categorical variables, the number of observations for each category (*n*) with the corresponding percentage (%) was presented. To compare paired categorical data, the McNemar test was used. *p* < 0.05 was considered statistically significant. The analysis was performed using STATISTICA PL 13.3 (TIBCO Software Inc., Palo Alto, CA, USA).

## 3. Results

### 3.1. General Characteristics of the Overall Study Group (Urgent Hospitalization with Both Preserved and Reduced LVEF)

The mean age in the study group (196 men and 66 women) was 67.6 ± 14.6 years, with a mean LVEF of 33.9 ± 14.8%. Dn HF was diagnosed in 74 (28.2%) subjects (Table 1.). Detailed characteristics are presented in Table 1. Six patients died during hospitalization. For all patients regardless of EF, ARNI/ACEI/ARB use rose from 63.3% at admission to 81.3% at discharge. Moreover, beta-blocker use increased from 70.6% (n = 185) to 92.6% (n = 237), MRA use increased from 43.1% (n = 113) to 75.8% (n = 194), and SGLT2i use increased from 30.1% (n = 79) to 75.0% (n = 192). These changes were all statistically relevant (*p* < 0.01). Therapy with ARNI/ACEI/ARB was optimized in 48.4% (n = 124) of the subjects, with optimization rates of 37.9% (n = 97), 40.2% (n = 103), and 44.1% (n = 113) for beta-blockers, MRAs, and SGLTi2s, respectively (Table 2.).

### 3.2. Characteristics of the HFrEF Group

In the study group, patients with reduced LVEF constituted 67.9% of the whole group (n = 178). They were predominantly male (n = 149; 83.7%), with a mean age of 65.2 ± 14.5 years and a mean LVEF of 25.2 ± 8.0%. Among them, 19 (10.7%) had a cardiac resynchronization therapy (CRT) device implanted, and 54 (30.5%) had an implantable cardioverter-defibrillator (ICD). Five patients from this subgroup died during hospitalization.

In the analysis for this subgroup, ARNI/ACEI/ARB use increased from 65.2% (n = 116) of the subjects at admission to 83.9% (n = 145) at discharge. Beta-blocker use increased from 71.9% (n = 128) to 94.2% (n = 168), MRA use increased from 46.6% (n = 83) to 83.8% (n = 149), and SGLT2i use increased from 37.1% (n = 65) to 84.4% (n = 150). ARNI/ACEI/ARB therapy was optimized in 53.7% (n = 93) of the subjects, with optimization rates of 39.3% (n = 70), 46.2% (n = 82), and 46.2% (n = 82) for beta-blockers, MRAs and SGLTi2, respectively. These changes were all statistically relevant (*p* < 0.01). However, only 38 (22.0%) of the patients achieved the 4-pillar GDMT of SGLT2i and ARNI/ACEI/ARB and BB and MRA in doses ≥ 50% (Table 3).

## 4. Discussion

Over 80% of ADHF patients in the HEROES registry were discharged on 4-pillar GDMT; however, most received these medications at suboptimal doses. Given the observational design of the registry, these findings should be interpreted with caution. The study is limited by potential confounding factors and the inability to establish causality, which underscores the need for careful consideration when drawing conclusions about treatment effectiveness and dosing strategies in this real-world population.

When compared to our population, coronary artery disease (CAD) and HF with ischemic etiology were diagnosed less frequently in the Victoria registry; this may be due to our study including patients with HF with preserved and mildly reduced ejection fractions (HFpEF and HFmrEF) [15]. In the Indian registry, ischemic etiology and CAD were reported in 69% and 71% of patients, respectively [16]; however, Grewal et al. reported ischemic HF a rate of 30.8%, similar to that of our study [17]. Likewise, in the STRONG-HF population, ischemic HF was reported in 48% of patients [5].

Regarding implantable devices, Green et al. reported that 34.8% and 9.9% of patients had ICDs and CRTs, respectively [15], which is comparable to our data. Surprisingly, Mebaza et al. [5] reported that only 1% of their patients had ICDs or CRTs, despite the fact that 85% of their patients had a history of HF, and the mean LVEF at baseline was 36.3%. This may be related to study’s inclusion criteria, which allowed for patients who could not be treated with full doses of GDMT.

With regard to other clinical tests, the STRONG-HF study reported that patients had lower mean concentrations of NTproBNP at screening (7110.7 ng/L) and at baseline (4025.6 ng/L) [5] than the HEROES population (at admission 9015 pg/mL). Another crucial parameter for the optimization of GDMT is blood pressure, which was higher in the STRONG-HF and Grewall et al. studies (123/74 mmHg and 129.8/78.1 mmHg, respectively) than in our study (at admission 129.7/79.2 mmHg, at discharge 116/72 mmHg) [5,15]. The incidence of CKD was also lower in the Indian registry (29%) [16] and the Grewall et al. study (21.7%) [15] than in our study (38.2%).

In the HEROES registry, the rate of GDMT implementation was higher than in the Victoria registry (66.8% vs. 81.2% for renin-angiotensin system inhibitors [RAASi], 75.1% vs. 92.6% for beta-blockers, and 44.9% vs. 75.8% for MRA) [15]. Moreover, in the Victoria population, up-titration was performed less commonly than in the HEROES study (23.3% vs. 48.3% for RAASi, 20.4% vs. 37.9% for beta-blockers, and 22.3% vs. 40.2% for MRA). It should be noted that, in our cohort, 31.3% of patients received intravenous inotropes, while this was less than 3% in the Victoria registry. The duration of hospitalization was also longer in our study (median of 9 days) than in the Victoria registry (median of 6 days) [15]. Regarding RAASi, our results are similar to those reported in previous studies. For instance, in the EPICAL2 study, 80.2% of patients were treated with ACE-I or ARB at discharge [18]. When compared to data from the GUIDE-IT trial, our results are closer to those observed in Canada (RAASi usage rate of 84.8%) than in the US (78.5%) [19].

We also observed that GDMT during ADHF hospitalization was optimized frequently (53%, 39.3%, 46.2%, and 46.2% for RAASi, beta-blockers, MRA, and SGLT2i, respectively), whereas down-titration was performed less frequently (11.5%, 16.3%, and 5.8% for RAASi, beta-blockers, and MRA, respectively). Our data are not consistent with the findings of Grewal et al., who reported that RAASi, beta-blockers, and MRA were up-tritiated (increased or initiated) in 25.5%, 39.6%, and 5.2% of patients, respectively, and down-titrated in 39.2%, 34.6%, and 42.6% of patients, respectively [17]. In the Victoria registry, down-titration rates were 13.3%, 9%, and 5% for RAASi, beta-blockers, and MRA, respectively [15]. These data illustrate the obstacles to GDMT implantation during the vulnerable phase of ADHF. In the STRONG-HF study, 64%, 36%, and 95% of patients before randomization were treated using RAASi, beta-blockers, and MRA, respectively [5]. It is worth mentioning that we achieved MRA optimization in a large proportion of patients, despite reports of MRA being the most difficult drug class to optimize due to common contraindications [20]. Notably, there was a high prevalence of GDMT usage (RAASi, beta-blockers, and MRA) at admission. Our results are most similar to those of the Get With the Guidelines Heart Failure Registry, in which 90.1%, 87.4%, and 25.2% of patients were initiated on beta-blockers, RAASi, and MRA, respectively [21].

The usage of ARNIs in the HEROES registry was comparable to that of the TRITATE-HF study (which involved patients with worsening HFrEF) [22], although the use of RAASi, beta-blockers, MRA, and SGLT2i at discharge were higher in our cohort. We also noted that SGLT2i therapy is still a neglected pillar of GDMT. Our SGLT2i results were similar to those of the EVOLUTION HF multi-national registry, which showed delayed initiation of novel GDMTs (SGLT2i and ARNI) [23]. On the other hand, we reported better results than Okoroike et al.’s study, in which only 6.6% of patients initiated SGLT2i therapy during hospitalization [24]. When our data were compared with those of the ESC Heart Failure Long-Term Registry, we noted that the prescription rates of RAASi, beta-blockers, and MRA at discharge were lower 10 years ago at 77%, 71.8%, and 55.3%, respectively [25].

Multiple studies have highlighted the importance of initiating GDMT as early as possible to reduce mortality and the need for hospitalization [5,26,27,28,29,30]. The up-titration of GDMT in outpatient settings after hospitalization is also equally important [1,5]. It has been reported previously that the discontinuation rates of ARNI, ACE-I/ARB, and MRA were approximately 10%, 15%, and 30%, respectively, with the main reason for discontinuation being chronic kidney disease (CKD) for RAASi [20,23], and hyperkaliemia and renal dysfunction for MRA [31].

We also observed an increased frequency of use of other drugs, such as digoxin (to control heart rate), when compared to the Indian registry; however, Onteddu et al. reported that ivabradine use was more frequent [16]. This observation may be due to a higher rate of atrial fibrillation in our cohort (56.5% vs. 16%) [16]. Furthermore, in the TRITATE-HF study, antihyperlipidemic drugs and ivabradine were used less frequently than in the HEROES study, but anticoagulants and digoxin were used more frequently [22]. Moreover, compared to patients hospitalized electively, optimization of RAASi therapy was significantly more frequent in those admitted for disease exacerbation (in whole group 33.1%, in HFrEF group 53.7%) [13]. These confirm that patients in a stable condition are less frequently considered candidates for treatment optimization [12].

Effective implementation of GDMT remains a critical goal in improving outcomes for HF patients in Poland. The findings from the HEROES registry highlight that, while initiation rates of GDMT are encouragingly high, achieving optimal dosing remains a significant challenge. To address this gap, clinical practice should focus on identifying and overcoming key barriers to up-titration, which may include concerns about drug tolerability, side effects, comorbidities, and patients’ clinical stability. Strategies such as structured follow-up visits, multidisciplinary HF clinics, and enhanced patient education can facilitate safe and timely dose escalation. In addition, increasing awareness and training among healthcare providers on the benefits and management of GDMT optimization could improve adherence to guidelines. Addressing system-level obstacles, including streamlined access to medications and better integration of outpatient care, is also essential. Ultimately, tailored approaches aimed at overcoming these barriers can help maximize the therapeutic benefit of GDMT, improving prognosis for HF patients across Poland.

### Limitations

To our knowledge, only one study has analyzed the up- and down-titration of GDMT in Polish patients with HFrEF who were hospitalized due to ADHF. The results of the current analysis should be interpreted in the context of several limitations. First, due to its observational registry design, some data regarding both admission and in-hospital characteristics were missing, although all data regarding the use of GDMT and LVEF were available for all patients in the analyzed group. Second, HEROES patients were recruited from selected centers, which might have affected the characteristics of the study population; the population reported in this study is not fully representative of the entire HF patient population, and therefore these results should not be generalized. Third, another limitation of our study was the relatively small size of the evaluated group, which depended on the availability of a known LVEF. The limited statistical power for subgroup analyses, and caution against overinterpreting results for low-prevalence subgroups (e.g., patients with CRT) should be also mentioned. Another limitation of this analysis is the lack of evaluation of the reasons for individual decisions on GDMT implementation and dosing, especially the influence of potential confounders like comorbidities and baseline disease severity. Although beyond the scope of this paper, it represents a valuable direction for further research aimed at better understanding therapeutic challenges in real-world practice. Other limitations of the HEROES study have also been previously presented [12].

## 5. Conclusions

In patients hospitalized due to ADHF in the HEROES study, the use of GDMT at discharge was notably higher than at admission. Among patients with reduced ejection fraction, over 80% received all classes of GDMT at discharge. Nevertheless, relatively few patients achieved target doses, highlighting the importance of ongoing efforts to optimize therapy doses in the outpatient setting.

## Figures and Tables

**Table 1:** Characteristics of the study group (all patients hospitalized for ADHF regardless of LVEF).

Demographic Characteristics	Study Group (n = 262) Mean ± SD; Median (Q1–Q3) or n (%)	
Age (years) ^262^	69.4 (61.3–77.9)	
Female ^262^	66 (25.2%)	
BMI (kg/m^2^) ^262^	28.4 (25.0–32.4)	
At least 1 HF hospitalization in the last 6 months ^168^	107 (63.7%)	
Number of hospitalizations due to ADHF in the last 6 months (0/1/2/3/4/5/6) ^262^	61 (23.3%)/59 (22.5%)/34 (13.0%)/9 (3.4%)/1 (3.8%)/2 (7.6%)/2 (7.6%)	
Prior diagnosis of HF ^262^	188 (71.8%)	
HF with ischemic etiology ^262^	89 (34.0%)	
LVEF (%) ^262^	30 (20.0–45.0)	
HFrEF/HFpEF/HFmrEF ^262^	178 (67.9%)/52 (19.8%)/32 (12.2%)	
Smoking (current/former/never) ^262^	109 (41.6%)/44 (16.8%)/109 (41.6%)	
In-hospital death ^262^	6 (2.3%)	
Length of hospital stay (days) ^260^	9 (6.0–14.0)	
**Clinical Status at Admission**		
NYHA Class I/II/III/IV ^262^	3 (1.61%)/20 (7.6%)/147 (56.1%)/92 (35.1%)	
Forester Classification: dry-warm/dry-cold/wet-warm/wet-cold ^262^	86 (32.8%)/157 (59.9%)/10 (3.8%)/9 (3.4%)	
Systolic blood pressure (mmHg) ^262^	130 (110.0–145.0)	
Diastolic blood pressure (mmHg) ^262^	80 (68.0–90.0)	
Heart rate (bpm) ^262^	85 (74.0–103.0)	
Reduced exercise tolerance ^262^	277 (67.2%)	
Dyspnea at rest ^262^	97 (37.0%)	
Orthopnea ^262^	123 (46.9%)	
Pulmonary rales ^262^	171 (65.3%)	
Peripheral edema ^262^	176 (67.2%)	
Hepatomegaly ^262^	27 (10.3%)	
Ascites ^262^	23 (8.8%)	
Elevated jugular venous pressure ^262^	59 (22.5%)	
Hepatojugular reflux ^262^	37 (14.1%)	
Third heart sound ^262^	10 (3.8%)	
Pleural effusion ^262^	81 (30.9%)	
**Clinical Status at Discharge**		
NYHA Class I/II/III/IV ^262^	22 (8.4%)/176 (67.2%)/58 (22.1%)/6 (2.3%)	
Forester Classification: dry-warm/dry-cold/wet-warm/wet-cold ^262^	213 (81.3%)/37 (14.1%)/9 (3.4%)/3 (1.1%)	
Systolic blood pressure (mmHg) ^257^	116 (105.0–125.0)	
Diastolic blood pressure (mmHg) ^260^	71 (63.0–80.0)	
Heart rate (bpm) ^261^	73 (66.0–80.0)	
Pulmonary rales ^262^	41 (15.6%)	
Peripheral edema ^262^	57 (21.8%)	
Hepatomegaly ^262^	7 (2.7%)	
Ascites ^262^	6 (2.3%)	
Elevated jugular venous pressure ^262^	14 (5.3%)	
Hepatojugular reflux ^262^	4 (1.5%)	
Third heart sound ^262^	3 (1.1%)	
Pleural effusion ^262^	40 (15.3%)	
**Laboratory Tests at Admission**		
Hemoglobin (g/dL) ^259^	13.2 (11.4–14.8)	
eGFR (mL/min/1.73 m^2^) ^241^	65.0 (41.0–82.0)	
Sodium (mmol/L) ^245^	139.0 (136.6–141.0)	
Potassium (mmol/L) ^243^	4.4 (4.1–4.8)	
NTproBNP (pg/mL) ^218^	5684 (2919–10,410)	
**Laboratory Tests at Discharge**		
eGFR (mL/min/1.73 m^2^) ^221^	62.0 (43.0–80.0)	
**Comorbidities**		
Coronary artery disease ^262^	114 (43.5%)	
Prior PCI ^262^	66 (25.2%)	
Coronary artery disease bypass graft ^262^	24 (9.2%)	
Arterial hypertension ^262^	180 (68.7%)	
Valvular intervention ^262^	20 (7.6%)	
Chronic obstructive pulmonary disease ^262^	21 (8.0%)	
Asthma ^262^	11 (4.2%)	
Chronic kidney disease ^262^	100 (38.2%)	
Dialysis ^262^	1 (0.4%)	
Depression ^262^	14 (5.3%)	
Cognitive dysfunction ^262^	6 (2.3%)	
Peripheral arterial disease ^262^	13 (5.0%)	
Severe liver insufficiency ^262^	1 (0.4%)	
Cancer ^262^	20 (7.6%)	
Prior myocardial infarction ^262^	84 (32.1%)	
Atrial fibrillation ^262^	148 (56.5%)	
Prior stroke ^262^	19 (7.3%)	
Prior TIA ^262^	6 (2.3%)	
Diabetes mellitus ^262^	99 (37.8%)	
Implanted CRT ^260^	21 (8.1%)	
Implanted ICD ^260^	58 (22.3%)	
**Medications/Interventions During Hospitalization**		
Vasoactive drugs ^259^	58 (22.4%)	
IV nitrates ^259^	53 (20.5%)	
Mechanical circulatory support ^259^	5 (1.9%)	
Electric cardioversion ^259^	10 (3.9%)	
Dialysis or ultrafiltration ^259^	3 (1.2%)	
Respiratory support ^259^	15 (5.8%)	
Vasoactive support ^259^		
Dobutamine ^259^	49 (18.9%)	
Dopamine ^259^	4 (5.4%)	
Milrinone ^259^	2 (0.8%)	
Levosimendan ^259^	3 (1.2%)	
Norepinephrine ^259^	11 (4.2%)	
Epinephrine ^259^	2 (0.8%)	
Vasopressin ^259^	0 (0%)	
Diuretics		
Furosemide ^261^	184 (71.0%)	
Torasemide ^261^	77 (29.7%)	
**Medications**	**At admission**	**At discharge**
Ivabradine ^259^	10 (3.8%)	21 (8.0%)
Diuretics ^261^	175 (67.6%)	360 (87.8%)
Digoxin ^259^	16 (6.1%)	28 (10.8%)
Statins ^259^	136 (52.5%)	186 (71.8%)
Antiplatelet ^259^	72 (27.8%)	92 (35.5%)
Anticoagulants ^259^	116 (44.9%)	153 (59.0%)
Dihydropyridine calcium blocker ^259^	36 (13.9%)	32 (12.4%)
Nondihydropyridine calcium blocker ^259^	0 (0.0%)	0 (0.0%)
Amiodarone ^259^	30 (11.6%)	34 (13.1%)
Other antiarrhythmics ^259^	4 (1.5%)	2 (0.7%)
Nitrates ^259^	2 (0.7%)	2 (0.7%)

Abbreviations: ADHF—acutely decompensated heart failure, BMI—body mass index, CRT—cardiac resynchronization therapy, eGFR—estimated glomerular filtration rate, HF—heart failure, HFmrEF—heart failure with mildly reduced ejection fraction, HFpEF—heart failure with preserved ejection fraction, HFrEF—heart failure with reduced ejection fraction, ICD—implantable cardioverter-defibrillator, IV—intravenous, LVEF—left ventricular ejection fraction, NTproBNP—N-terminal pro-brain natriuretic peptide, NYHA—New York Heart Association, PCI—percutaneous coronary interventions, SD—standard deviation, TIA—transient ischemic attack. Superscripted number refers to the number of available data.

**Table 2:** Medication use and titration in the study group (all patients regardless of LVEF).

Medication Class ^262^	Usage at Admission n (%)	Usage at Discharge n (%)	Dose at Admission		Dose at Discharge		Up-Titration/ Down-Titration n (%)
			% of Target Dose Category	n (%)	% of Target Dose Category	n (%)	
ARNI	34 (13.0)	71 (27.7)	1–49 50–99 100	14 (5.3) 11 (4.2) 9 (3.4)	1–49 50–99 100	38 (14.8) 25 (9.9) 8 (3.1)	49 (19.1)/ 3 (1.2)
ACEI	106 (40.5)	121 (47.2)	1–49 50–99 100	44 (16.8) 41 (15.6) 21 (8.0)	1–49 50–99 100	66 (25.8) 39 (15) 15 (5.9)	65 (25.4)/ 28 (10.9)
ARB	26 (9.9)	16 (6.3)	1–49 50–99 100	10 (3.8) 9 (3.4) 7 (2.7)	1–49 50–99 100	11 (4.4) 4 (1.6) 0 (0)	10 (3.9)/ 5 (2.0)
Beta-blocker	185 (70.6)	237 (92.6)	1–49 50–99 100	91 (34.7) 65 (24.8) 29 (11.1)	1–49 50–99 100	110 (43.0) 97 (37.9) 30 (11.7)	97 (37.9)/ 38 (14.8)
MRA	113 (43.1)	194 (75.8)	1–49 50–99 100	0 (0) 75 (28.6) 38 (14.5)	1–49 50–99 100	1 (0.4) 126 (49.2) 67 (26.2)	103 (40.2)/ 15 (5.9)
SGLT2i	79 (30.1)	192 (75.0)	100	79 (30.1)	100	192 (75.0)	113 (44.1)

Abbreviations: ACE-I—angiotensin-converting enzyme inhibitor, ARB—angiotensin receptor blockers, ARNI—angiotensin receptor-neprilysin inhibitor, LVEF—left ventricular ejection fraction, MRA—mineralocorticoid receptor antagonist, SGLT2i—sodium glucose cotransporter 2 inhibitor. Superscripted number refers to the number of available data.

**Table 3:** Medication use and titration in the HFrEF subgroup.

Medication Class ^178^	Usage at Admission n (%)	Usage at Discharge n (%)	Dose at Admission		Dose at Discharge		Up-Titration/ Down-Titration n (%)
			% of Target Dose Category	n (%)	% of Target Dose Category	n (%)	
ARNI	34 (19.1)	70 (40.5)	1–49 50–99 100	14 (7.9) 11 (6.2) 9 (5.0)	1–49 50–99 100	37 (21.4) 25 (14.5) 8 (4.6)	48 (27.7)/ 3 (1.7)
ACEI	68 (38.2)	69 (39.9)	1–49 50–99 100	31 (17.4) 25 (14.0) 12 (6.7)	1–49 50–99 100	38 (22.0) 24 (13.9) 7 (4.0)	38 (22.0)/16 (9.2)
ARB	14 (7.9)	6 (3.5)	1–49 50–99 100	4 (2.2) 5 (2.8) 5 (2.8)	1–49 50–99% 100	5 (2.9) 1 (0.6) 0 (0)	7 (4.0)/ 1 (0.6)
Beta-blocker	128 (71.9)	163 (94.2)	1–49 50–99 100	65 (36.5) 42 (23.6) 21 (11.8)	1–49 50–99 100	69 (39.9) 72 (41.6) 22 (12.7)	68 (39.3)/29 (16.3)
MRA	83 (46.6)	145 (83.8)	1–49 50–99 100	0 (0%) 53 (29.8) 30 (16.9)	1–49 50–99 100	1 (0.6) 90 (52.0) 54 (31.2)	80 (46.2)/10 (5.8)
SGLT2i	66 (37.1)	146 (84.4)	100	66 (37.1)	100	146 (84.4)	80 (46.2)

Abbreviations: ACE-I—angiotensin converting enzyme inhibitor, ARB—angiotensin receptor blockers, ARNI—angiotensin receptor-neprilysin inhibitor, HFrEF—heart failure with reduced ejection fraction, MRA—mineralocorticoid receptor antagonist, SGLT2i—sodium glucose cotransporter 2 inhibitor. Superscripted number refers to the number of available data.

## Data Availability

Data are available to all members of the Polish Cardiac Society. https://doi.org/10.60941/JVH1-5190. For other interested parties, data are not publicly accessible but may be provided upon reasonable request and with the approval of the principal investigator.

## Abbreviations

The following abbreviations are used in this manuscript:

ACE-I	angiotensin-converting enzyme inhibitor
ADHF	acutely decompensated heart failure
ARB	angiotensin receptor blockers
ARNI	angiotensin receptor-neprilysin inhibitor
BMI	body mass index
CAD	coronary artery disease
CKD	chronic kidney disease
CRT	cardiac resynchronization therapy
dnHF	de novo heart failure
eGFR	estimated glomerular filtration rate
GDMT	guideline-directed medical therapy
HEROES study	HEart failuRe ObsErvational Study
HF	heart failure
HFmrEF	heart failure with mildly reduced ejection fraction
HFpEF	heart failure with preserved ejection fraction
HFrEF	heart failure with reduced ejection fraction
HR	heart rate
ICD	implantable cardioverter-defibrillator
IV	intravenous
LVEF	left ventricular ejection fraction
MRA	mineralocorticoid receptor antagonist
NTproBNP	N-terminal pro-brain natriuretic peptide
NYHA	New York Heart Association
PCI	percutaneous coronary interventions
RAASi	renin-angiotensin-aldosterone system inhibitors
SGLT2i	sodium glucose cotransporter 2 inhibitor
STRONG-HF	Safety, Tolerability and Efficacy of Rapid Optimization of Heart Failure
SD	standard deviation
TIA	transient ischemic attack
